# A Doubly Bridged Bis(phenylethynyl)benzene: Different from a Twisted Tolan

**DOI:** 10.1002/chem.202002552

**Published:** 2020-11-24

**Authors:** Manuel Hodecker, Yury Kozhemyakin, Svenja Weigold, Frank Rominger, Jan Freudenberg, Andreas Dreuw, Uwe H. F. Bunz

**Affiliations:** ^1^ Interdiziplinäres Zentrum für Wissenschaftliches Rechnen Ruprecht-Karls-Universität Heidelberg Im Neuenheimer Feld 205 69120 Heidelberg Germany; ^2^ Organisch-Chemisches Institut Ruprecht-Karls-Universität Heidelberg Im Neuenheimer Feld 270 69120 Heidelberg Germany

**Keywords:** bis(phenylethnynyl)benzene, conformational analysis, tolan, tolanophanes

## Abstract

The synthesis of a doubly bridged 1,4‐bis(phenylethynyl)benzene is reported. The target displays photophysical properties, distinctly different from that of its congeners, the singly bridged tolans. Quantum‐chemical calculations suggest a lack of planarization of the bridged bis(phenylethynyl)benzene in the first excited state.

## Graphical abstract

**Two are better than one?** This work reports the synthesis and properties of a twofold bridged bis(phenylethynyl)benzene, which, due to its two tethers, does not planarize in the excited state in contrast to its mono‐bridged tolan counterpart.

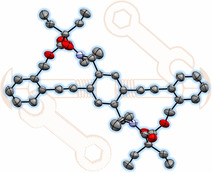

Diphenylacetylene (tolan, blue substructure in Figure 1) is a fundamental hydrocarbon chromophore. It is distinct from other chromophores as its electronic properties are dramatically influenced by its *rotational* conformation, that is, the twist of the phenyl rings to each other. We and others have investigated the fixation of the dihedral angle of the two phenyl rings using steric pressure^[1]^ or suitable tethers.[^2^, ^3^, ^4^, ^5^] Depending on its twist angle, tolans display substantially different excited‐state behavior. Planar tolans absorb at around 300 nm, while twisted derivatives absorb at shorter wavelengths (around 280 nm). In contrast to planar ones, twisted tolans (Figure 1, **A, B1**)[^5c^, ^5d^] display strong phosphorescence with long lifetimes at low temperatures (e.g. *τ*=4 s for **A**).

**Figure 1:**
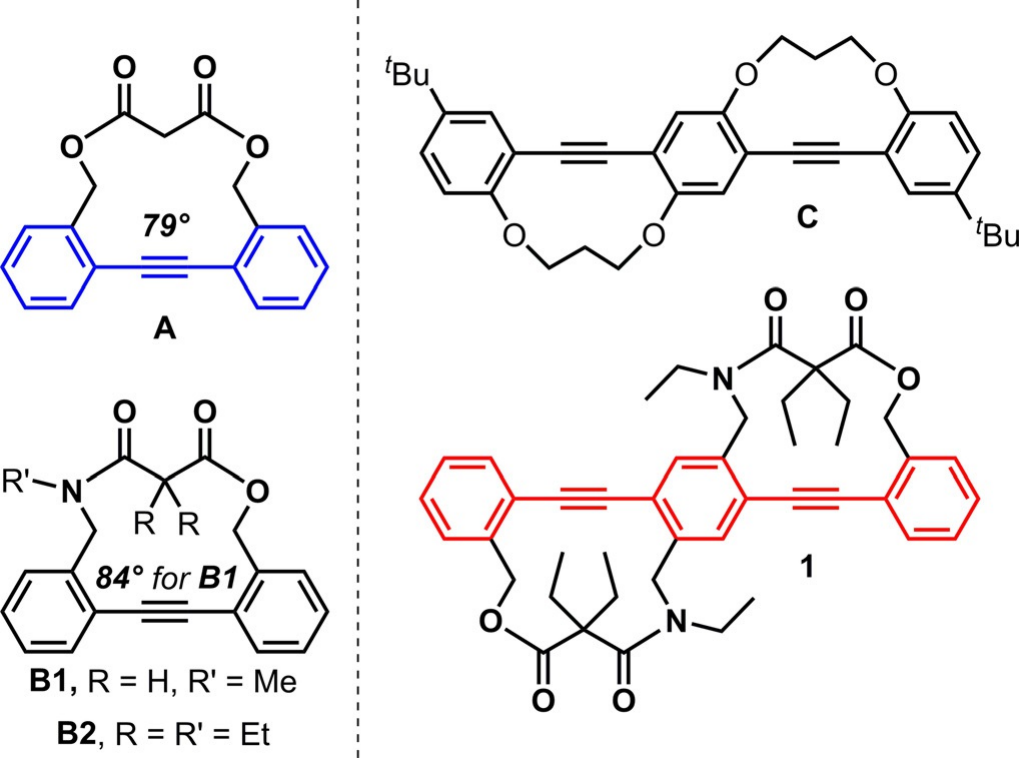
Singly bridged synthesized tolanes and their twist angles (**A**, **B1**) as well as doubly bridged bis(phenylethynyl)benzenes **C** and **1**. Substructures highlighted: Tolan (blue), bis(phenylethynyl)benzene **BPEP** (red).

1,4‐Bis(phenylethynyl)benzene (**BPEB**, red substructure in Figure 1) is obtained by π‐extending tolan with an additional phenylethynyl group in *para*‐position. In the fully planar conformation, this leads to energetically lower‐lying excited states, resulting in red‐shifted absorption and emission spectra. Similar to tolan, the first excited state (ππ*) of **BPEP** is planar and quinoidal‐cumulenic in nature, and from this it can either fluoresce or internally convert into a dark state (πσ*), with one triple bond exhibiting a *trans*‐stilbene like structure (termed *trans*‐bent), whose population is a prerequisite for phosphorescence (see Supporting Information Chart C1 for structures).^[6]^ The twisting angles, that is, the relative orientation of the three phenyl rings to each other, influences the optical properties as illustrated by **BPEP**’s wavelength dependent emission.^[7]^ Crisp and Bubner synthesized **C** with a constrained π‐system.^[3c]^ Narrow absorption bands with increased *ϵ* resulted, but this has not been rationalized in terms of the twist angles and electronic transitions. At large twist angles, **BPEP** should display optoelectronics similar to that of twisted tolans. We here report a twisted bis(phenylethynyl)benzene **1** and its experimental and quantum‐chemical photophysical properties. As tether we chose a maleic acid monoamide moiety, as the twist angle of tolan **B** was increased compared to that of the maleic ester derivative **A** in the respective crystal structure.

Starting from bis(bromomethyl)diiodobenzene **2** ^[8]^ (Scheme 1), its reaction with ethyl amine and reaction with *t*‐Boc_2_O furnishes protected amine **3**, which is coupled to benzylic alcohol **4** ^[9]^ to give the open precursor **5** (yield 68 %) over three steps. Deprotection (≈97 %) and ring closure by amide/ester formation results in the target **1** in 60 % yield. The ethyl groups on the nitrogen and the malonic ester provide solubility in DCM, THF or hexane and allow chromatographic purification of **1**.

**Scheme 1:**
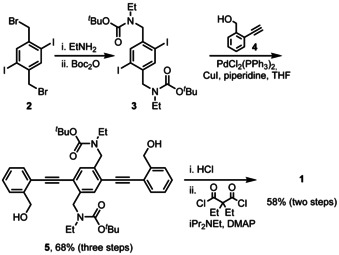
Synthesis of the target compound **1**.

Figure 2 displays the UV/Vis spectrum of the twisted bis(phenylethynyl)benzene **1** in solution at ambient temperature and the emission spectra at ambient temperature and in an EPA‐glass at 77 K. The UV/Vis spectrum is a bit broadened, similar to that of the planar, unsubstituted **BPEB**.^[10]^ The emission spectra at room temperature and at 77 K are identical with the exception of a feature localized at around 511 nm, that is, weak phosphorescence, red‐shifted when compared to that of bridged tolans. Tolans display a strong emission at 450 nm—suggesting that the emission at 511 nm is phosphorescence out of the bis(phenylethynyl)benzene unit (for a comparison see Figure S6, Supporting Information).

**Figure 2:**
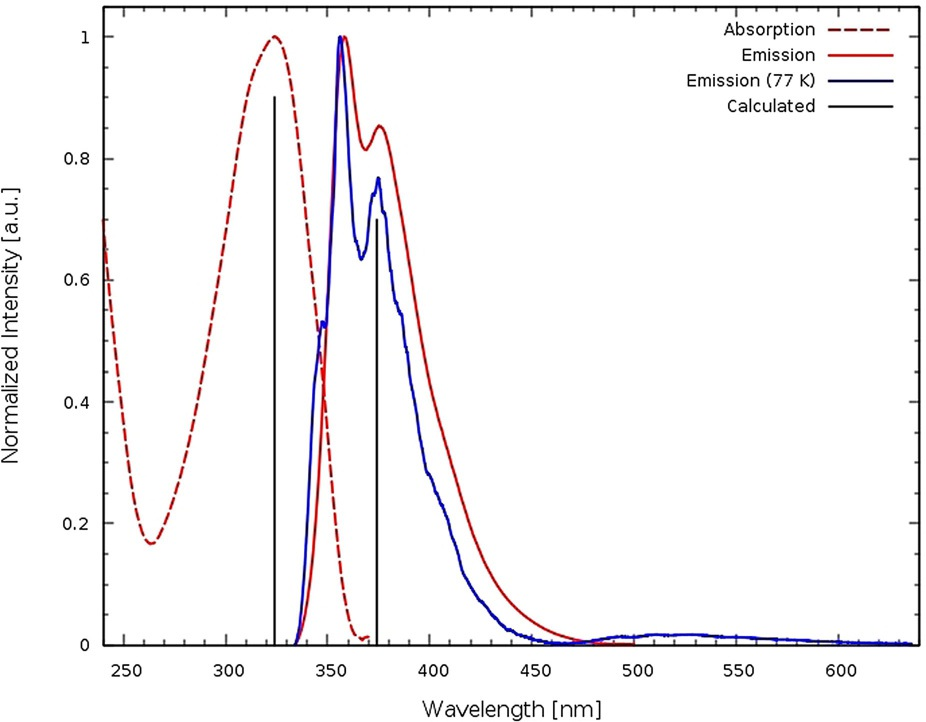
UV/Vis absorption (dashed) and emission spectrum (solid) of the doubly bridged bis(phenylethynyl)benzene **1** in *n*‐hexane at room temperature (red) and emission spectrum in a frozen EPA (Et_2_O/isopentane/EtOH 5:5:2 v/v/v) glass (blue) at 77 K after excitation at 324 nm; *λ*_max_ absorption is at 325 nm (*ϵ*=3.2×10^4^ L mol^−1^ cm^−1^) and *λ*_max_ emission is at 356 nm and 511 nm. The vertical black bars correspond to calculated excitation energies.

The fluorescence quantum yield of **1** is 0.6 in cyclohexane with a lifetime of 0.5 ns at room temperature. When going to EPA at 77 K, the fluorescence lifetime even increases to 0.8 ns. The intensity of the phosphorescence signal was too low to allow lifetime determination. **1** displays some features of a twisted system but these spectroscopic signatures are weak.

Figure 3 shows the unit cell of **1** containing two independent half‐molecules. The twist angles (i.e., the angles spanned by the averaged phenyl ring plane normal vectors) amount to 40° and 44°, respectively, and are thus lower than the angles observed in **A** and **B1**, where they are 79° and 84°, respectively.[^5c^, ^5d^] The interaction between the two benzene rings is quantified as overlap of the adjacent atomic p orbitals; it is proportional to (cos α)^2^ with values of 0.59 at α=40°, and 0.52 at 44° twist, while at 79° twist the overlap is only 0.04. Hence, the π‐conjugation in **5** is only somewhat reduced.

**Figure 3:**
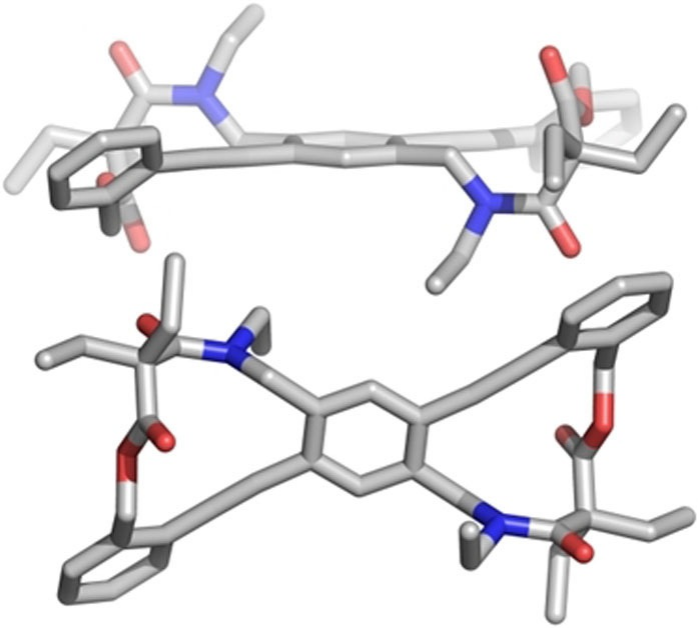
Single‐crystal structure of compound **1**. Two independent half molecules are present in the asymmetric unit cell, both on a crystallographic inversion center. The twist angles are 40° and 44°, respectively. Hydrogen atoms and solvent molecules of chloroform are omitted for clarity.

Attempts to obtain a bis(phenylethynyl)benzene derivative of **A** with two malonic acid ester bridges, that is, where the two NEt groups and the CEt_2_ group of **1** are replaced by oxygen atoms and methylene groups, respectively, were hindered both by the poor solubility and the trace yields of the cyclization product.

Twist angles (**A**: 79°, **1**: 40°, 44°) depend on the exact nature of the linker between the arene rings as suggested by quantum‐chemical calculations (DFT/B3LYP/def2‐TZVP level): The twisted diphenylacetylene **B2** with the linker of **1** revealed an angle of 35° only (see Figure 1). Thus, different linkers enforce different twist angles, which determine the electronic properties of the species.

Quantum‐chemical calculations using the Gaussian 16 program package^[11]^ have been performed to investigate and understand the photophysical behavior of compound **1**. Starting from the crystal structure, the ground‐state geometry was optimized in the gas phase at the DFT/B3LYP/def2‐TZVP level of theory.[^12^, ^13^, ^14^] Analysis of the harmonic frequencies confirmed the minimum nature of the stationary point. Further conformers with different torsion angles are not expected to contribute to the electronic spectrum (see Supporting Information). The twist angles exhibit values of about 35° each, in agreement with the experimental angle of 40°/44°. Grimme's dispersion correction (DFT‐D3)^[15]^ is of minor importance, changing the torsion angles only by about 1°. At this geometry, the UV/Vis absorption spectrum was simulated at the linear response time‐dependent density functional theory (TDDFT) level^[16]^ using the CAM‐B3LYP functional^[17]^ and the same basis set (def2‐TZVP), a methodology that gives excellent results for excited states of aromatic organic compounds.^[18]^ The calculated first excitation energy (3.83 eV, 324 nm) is in excellent agreement with the experiment (see first vertical bar in Figure 2). The S_1_ excited state corresponds to a typical HOMO–LUMO (π–π*) transition, see Figure S4. More vertical excitation energies and their oscillator strengths are shown in Table S2 in the Supporting Information.

The torsion angles of **1** are much smaller than the one **A** and **B1**, allowing for π‐conjugation manifesting itself in the absorption spectrum, red‐shifted compared to that of bridged and unbridged tolans.[^5^, ^7^] The peak of **1** is sharper compared to the one of bridged tolan **A**, however, compared to other non‐bridged bis(phenylethynyl)benzenes it is broader. This scales with the twist angles in the order of **BPEB** (planar)<**1**<**A**.

To investigate the emission properties of **1**, the geometry of the bright S_1_ state has been optimized at the TDDFT/CAM‐B3LYP/def2‐SVP level, revealing torsion angles of about 25°. Full planarization does not occur (for a comparison of the geometries, see Figure S5, Supporting Information). The same is true for the diphenylacetylene **B2** with the linker of **1**, where a torsion angle of 20° was calculated for the S_1_ equilibrium geometry. At this geometry, the S_1_ state of **1** is still the bright state with an emission energy of 3.32 eV (374 nm) to the S_0_ ground state, in good agreement with the experimental value of 356 nm (see second vertical bar in Figure 2). Furthermore, the lowest triplet state T_1_ has been optimized as the open‐shell ground state at the B3LYP/def2‐TZVP level as well as using TDDFT within the Tamm–Dancoff approximation^[19]^ (CAM‐B3LYP/def2‐SVP) taking the closed‐shell singlet as reference. The former approach resulted in a geometry with a twist angle of 24° and an energy difference to the S_0_ of 644 nm, whereas the latter yielded about 27° and an energy difference of 2.07 eV (600 nm), both in fairly good agreement to the broad experimental emission band between 500 and 600 nm at 77 K. Calculated emissions are lower in energy (red‐shifted) compared to the experimental emission energies, as vibrational effects were not taken into account in our simulation.

To summarize, when going from a singly bridged tolan to a doubly bridged 1,4‐bis(phenylethynyl)benzene, the photophysical behavior is markedly changed. While bridged tolans **A** and **B1** show broad absorption peaks and strong phosphorescence at 77 K, the absorption of its doubly‐bridged homologue **1** is red‐shifted and relatively sharp; phosphorescence is barely observable at 77 K. The twisted tolans **A** and **B1** planarize in the excited state, **1** does not, indicating that conjugation as the driving force is not sufficient in this case.

In addition, *trans*‐bent (i.e., *trans*‐stilbene‐like, biradical) structures, vital for triplet emission after excitation,^[6b]^ are unimportant, since the phosphorescence yield is very low. However, this will be investigated in more detail in a separate contribution.^[20]^

## Experimental Section

8,8,10,22,22,24‐Hexaethyl‐13,14,27,28‐tetradehydro‐10,11,24,25‐tetrahydro‐5 H,7*H*‐dibenzo[k,k′]benzo[1,2‐g:4,5‐g′]bis[1,5]oxazacyclotridecine‐7,9,21,23(8 H,19 H,22H)‐tetrone (1): A solution of HCl in Et_2_O (2 m, 16 mL) was added to a suspension of 5 (5.39 g, 8.25 mmol) in CH_2_Cl_2_ (66 mL) and MeOH (33 mL). After stirring for 3 days, the reaction mixture was diluted with Et_2_O (45 mL). The precipitate was collected by filtration and washed with CH_2_Cl_2_ (3×30 mL) to yield the deprotected bisammonium salt as a yellowish solid (4.26 g, 8.10 mmol, 98 %). A solution of NaOH (642 mg, 16.1 mmol, 4.0 equiv) in water (32 mL) was added to a fraction of the collected solid (2.11 g, 4.01 mmol, 1.0 equiv) suspended in CHCl_3_ (170 mL). After stirring for 5 min two clear phases formed. The aqueous layer was extracted with CHCl_3_ (1×50 mL) and combined with the organic phase, which was washed with brine (1×50 mL) and dried over MgSO_4_. Evaporation of volatiles in vacuo afforded the free diamine (1.77 g, 3.91 mmol, 97 %) as a beige solid, which was directly used in the next step. Under common Schlenk conditions, a solution of diethylmalonyl dichloride (1.42 mL, 8.27 mmol, 4.0 equiv) in dry CH_2_Cl_2_ (100 mL) was added during 5 h to a suspension of the crude diamine (936 mg, 2.07 mmol, 1.0 equiv), *i*Pr_2_NEt (1.45 mL, 8.27 mmol, 4.0 equiv), 4‐(dimethylamino)pyridine (505 mg, 4.14 mmol, 2.0 equiv) and NaHCO_3_ (1.39 g, 16.5 mmol, 8.0 equiv) in dry CH_2_Cl_2_ (1.0 L). After stirring for an additional 48 h, H_2_O (100 mL) was added and the aqueous phase was extracted with CH_2_Cl_2_ (2×50 mL). The combined organic layers were washed with saturated aq. NaHCO_3_ solution (100 mL), brine (100 mL) and dried over MgSO_4_. Purification via column chromatography (silica gel; CH_2_Cl_2_/ethyl acetate 30:1, v/v) yielded 1 as colorless solid. Yield: 864 mg (1.23 mmol, 60 %). *R*_f_=0.43 (petroleum ether/ethyl acetate 1:1, v/v); m.p.=349 °C (decomposition without melting); ^1^H NMR (CDCl_3_, 600 MHz): *δ*=0.72–0.89 (m; 12 H), 0.92–1.04 (m; 6 H), 1.79–2.11 (m; 8 H), 2.77–2.91 (m; 1 H), 3.24 (br s; 2 H), 3.48–3.64 (m; 1 H), 3.93–4.04 (m; 1 H), 4.44–4.79 (m; 2 H), 4.95‐5‐45 (m; 5 H), 7.31–7.41 (m; 6 H), 7.49–7.62 (m; 4 H) ppm; ^13^C NMR (CDCl_3_, 150 MHz): *δ*=8.0, 8.2, 12.2, 14.1, 22.3, 26.4, 49.3, 49.5, 50.0, 50.2, 67.2, 67.4, 90.4, 92.8, 93.1, 93.9, 94.2, 123.7, 123.8, 128.9, 129.0, 130.1, 132.5, 132.9, 135.5, 136.1, 136.3, 136.5, 137.3, 137.9, 138.3, 169.9, 170.3, 173.8, 174.3 ppm “both 1H and 13C NMR spectra show a complex mixture due to aggregates. The possibility of geometric isomer formation in solution can not totally be excluded.”; IR: $\tilde{\nu }$ =2968, 2877, 1716, 1629, 1507, 1398, 1300, 1208, 1132, 983, 746, 664 cm^−1^; HRMS (ESI^+^) *m*/*z*: [M+Na]^+^ calcd for C_44_H_48_N_2_NaO_6_^+^ : 723.3405; found: 723.3413, correct isotope distribution. Crystal data for 1: sum formula: C_46_H_50_Cl_6_N_2_O_6_, formula weight: 939.58 g mol^−1^, colorless crystal (little brick), obtained by slow diffusion of pentane into a CHCl_3_ solution of 1, dimensions 0.113×0.057×0.048 mm^3^, triclinic crystal system, space group *P* $\bar{1}$ , *Z=*2, *a=*9.6500(4) Å, *b=*15.8327(6) Å, *c=*16.2694(6) Å, *α*=70.925(3)°, *β*=85.317(3)°, *γ*=88.315(3)°, *V=*2341.39(16) Å^3^, *ρ*=1.333 g cm^−3^, *T=*100(2) K, *θ*_max_=55.084°, radiation Cu_Kα_, *λ*=1.54178 Å, 22 606 reflections measured, 5840 unique (*R_int_=*0.00712), 3959 observed (*I*>2*σ*(*I*)), final residual values *R*_1_(*F*)=0.061, *wR*(*F* ^2^)=0.150 for observed reflections.

Deposition Number(s) 2003158 contain(s) the supplementary crystallographic data for this paper. These data are provided free of charge by the joint Cambridge Crystallographic Data Centre and Fachinformationszentrum Karlsruhe Access Structures service www.ccdc.cam.ac.uk/structures.

## Conflict of interest

The authors declare no conflict of interest.

## Supporting information

As a service to our authors and readers, this journal provides supporting information supplied by the authors. Such materials are peer reviewed and may be re‐organized for online delivery, but are not copy‐edited or typeset. Technical support issues arising from supporting information (other than missing files) should be addressed to the authors.

Supplementary
