# Supplementary material for: A Doubly Bridged Bis(phenylethynyl)benzene: Different from a Twisted Tolan
Source: Chemistry. 2020 Nov 24;26(71):16990–3. doi: 10.1002/chem.202002552 (PMC7839586; doi:10.1002/chem.202002552)
Supplement: Supplementary file 1 — Supplementary [file CHEM-26-16990-s001.pdf]

Chemistry–A European Journal

Supporting Information

A Doubly Bridged Bis(phenylethynyl)benzene: Different from a Twisted Tolan

Manuel Hodecker,[a] Yury Kozhemyakin,[b] Svenja Weigold,[b] Frank Rominger,[b] Jan Freudenberg,*[b] Andreas Dreuw,*[a] and Uwe H. F. Bunz*[b]

List of Contents for Supporting Information

1. General Experimental Methods 2

2. Synthetic Procedures 3

3. NMR Spectra 4

4. Single Crystal Structure of Compound 1 7

5. Computational Studies 8

6. Comparison of Optical Spectra 20

7. Illustration of Excited States of BPEP 21

8. References 22

1. General Experimental Methods

All **reagents and solvents** were obtained from Fisher Scientific, ABCR, Acros Organics, Sigma-Aldrich, Merck or from the chemical shop of the Organic Chemistry Institute of Heidelberg University, and were used as obtained. Deuterated solvents were purchased from Deutero GmbH. Absolute solvents were dried using a MB SPS-800 solvent purification system. Preparation of oxygen and moisture sensitive materials was carried out in heat gun dried glassware under an atmosphere of nitrogen employing common Schlenk techniques. Degassing was performed by bubbling a stream of nitrogen through the solution for a minimum of 30 minutes or by repetitive freeze-pump-thaw cycles. For **slow addition of solutions**, a syringe pump (BBraun Perfusor VII) was employed. **Column chromatography** was performed using silica gel purchased from Sigma-Aldrich (technical grade, particle size: 0.063-0.200 mm) or from Macherey-Nagel GmbH & Co. KG, Düren (Germany) (particle size: 0.040-0.063 mm). **Nuclear magnetic resonance spectra** (^{1}H, ^{13}C) were recorded at room temperature (unless otherwise stated) on a Bruker Avance III 3000, Bruker Avance III 500 or Bruker Avance III 600 a t the NMR Spectroscopy Facility of Heidelberg University. Processing of the acquired data was done using Bruker TopSpin 3.2 or ACD/Spectrus Processor 2015 Pack 2. Chemical shifts (δ) are reported in parts per million (ppm) and referenced internally to the solvent signal.[1] **Melting points** were determined in glass capillaries with a Melting Point Apparatus MEL-TEMP (Electrothermal, Rochford, UK) and reported uncorrected to normal conditions.
Infrared (IR) spectra are reported in wavenumbers (cm^{-1}) and were recorded neat on a JASCO FT/IR-4100 spectrometer equipped with GladiATR attenuated total reflectance accessory from Pike Technologies. Data was handld using Jasco Spectra Manager 1.5 or 2.0. **High resolution mass spectra (HRMS)** were obtained by electrospray ionization (ESI), direct analysis in real time (DART) or electron ionization (EI) on a Bruker ApexQe hybrid 9.4 TFT-ICR-MS at the Mass Spectrometry Facility of Heidelberg University. **Elemental Analysis** was performed on an Elementar vario MICRO cube at the Microanalytical Laboratory of Heidelberg University. **Single crystal structure analysis** was performed at the Crystallography Facility of Heidelberg University on a Bruker APEX-II Quazar or a STOE Stradivari. Data was processed and visualized with Mercury 3.2 / 4.0 or PyMOL 1.3 software. Supplementary crystallographic data can be obtained free of charge from The Cambridge Crystallographic Data Centre via www.ccdc.cam.ac.uk/data_request/cif. **UV-Vis absorption spectra** were recorded at room temperature on a JASCO UV-VIS V-660 or V-670 spectrometer and at 94 K on a Agilent Cary 5000 UV-Vis-NIR spectrophotometer with a cryostat cell and a West 6100plus temperature control unit controlled by the Cary WinUV software. **Emission spectra** were recorded on a Jasco FP6500 spectrometer at room temperature in Quartz SUPRASIL® high precision cells with light path 10x10mm from Hellma Analytics. Low temperature measurements were conducted in epa (diethyl ether/iso-pentane/ethanol 5:5:2, v/v) as it forms a transparent rigid glass at liquid nitrogen temperatures. **Fluorescence lifetimes** were measured using a Horiba FluoroCube equipped with a TBX picosecond photon detection module. Samples were excited by Horiba laser light sources: NanoLED-290 (290 nm) for fluorescence and SpectraLED-295 (300 nm) for phosphorescence. Phosphorescence lifetimes were measured at 77 K in a Jasco LPH-140 cell adjusted to a Horiba FluoroCube. Data were evaluated with the Horiba DAS6 software. **Fluorescence quantum yields** φ were determined using either the absolute method[2] on a PTI Quantamaster 40 equipped with an Ulbricht sphere or utilizing the comparative method with L-Tryptophan in distilled water as a reference system (φ_{ref}=0.14[3]). At least data from four different concentrations were used.

2. Synthetic Procedures

Dibromide **2** was synthesized starting from commercially available terephthalaldehyde using a published synthetic sequence.[4] The yield on the iodination step was improved from 23% to 77% using sodium periodate instead of iodic acid. Benzyl alcohol **4** was synthesized according to procedure reported in literature.[5]

Di-*tert*-butyl ((2,5-diiodo-1,4-phenylene)bis(methylene))bis(ethylcarbamate) (3).
Dibromide **2** (7.30 g, 14.2 mmol, 1.00 eq.) was dissolved in chloroform (890 mL) and ethylamine (70% in H_2O, 380 mL, excess) was added. The reaction mixture was stirred overnight at rt. Solvents were concentrated under reduced pressure and the aqueous phase was extracted with chloroform (3x150 mL). The organic layers were combined, washed with aqueous NaOH (150 mL, 1 M) and H_2O (150 mL) and subsequently dried over sodium sulfate. The diamine, obtained as a colorless solid (6.03 g, 13.6 mmol, 96%) was used in the next step without further purification. The diamine intermediate (12.7 g, 28.7 mmol, 1.00 eq.) was dissolved in dry THF (100 mL). Then di-*tert*-butyl dicarbonate (14.0 mL, 60.5 mmol, 2.10 eq.) was added in one portion and the reaction mixture was stirred for 1 h. After evaporation of the solvent under reduced pressure, the crude product was purified by column chromatography (SiO_2, petroleum ether:ethyl acetate 30:1, v/v) to yield a colorless solid (18.4 g, 28.5 mmol, 99%, 95% over two steps). R_f = 0.72 (SiO_2, DCM:MeOH 10:1, v/v); m.p. = 95 °C; ^{1}H NMR ($CDCl_3$, 600 MHz): δ = 7.51 (s, 2H), 4.32 (d, *J*=48.0 Hz, 4H), 3.27 (d, *J*=70.8 Hz, 4H), 1.45 (d, *J*=75 Hz, 18H), 1.11 (s, 6H) ppm. ^{13}C NMR ($CDCl_3$, 150 MHz): δ = 156.0, 155.4, 141.3, 141.0, 138.1, 137.8, 98.4, 98.1, 97.8, 97.6, 80.2, 54.7, 54.1, 42.3, 28.6, 13.7, 13.5 ppm; both NMR spectra show a complex mixture of tautomers; IR: $\tilde{\nu}$ = 2977, 2927, 1689, 1478, 1451, 1428, 1406, 1364, 1320, 1252, 1144, 1073, 1032, 980, 950, 879, 758, 679, 419 cm^{-1}. HRMS (DART+) m/z: $[M+NH_4]^+$: calcd. for $C_{22}H_{38}I_2N_3O_4$: 662.0946; found 662.0941, correct isotope distribution; elemental analysis calcd. for $C_{22}H_{34}I_2N_2O_4$: C: 41.01%, H: 5.32%, N: 4.35%, found: C: 41.00%, H: 5.04%, N: 4.41%.

Di-*tert*-butyl ((2,5-bis((2-(hydroxymethyl)phenyl)ethynyl)-1,4-phenylene)bis(methylene))bis(ethylcarbamate) (5).
The reaction was performed under standard Schlenk conditions. A degassed solution of **3** (2.01 g, 3.12 mmol, 1.0 eq), $PdCl_2(PPh_3)_2$ (109 mg, 156 µmol, 5 mol%) and CuI (59.0 mg, 312 µmol, 10 mol%) in THF (18 mL) and piperidine (9 mL) was added to nitrogen purged **3** (824 mg, 6.24 mmol, 2.0 eq) and allowed to stir over night. Afterwards solvent was changed to CH_2Cl_2 and coated onto Celite by evaporation of the solvend under reduced pressure. Purification by column chromatography (silica gel; CH_2Cl_2/methanol gradient from 20:1 to 2:1, v/v) followed by washing with methanol (4×20 mL) yielded **5** as a beige solid. Yield: 1.47 g (2.26 mmol, 72%, 68% over three steps starting from **2**). R_f = 0.27 (SiO_2, petroleum ether:ethyl acetate 1:1, v/v); m.p. = 215 °C (dec.); ^{1}H NMR (pyridine-d_5, 600 MHz): δ = 8.07 (d, *J* = 7.2 Hz, 2H), 7.85 (bs, 2H), 7.70 (d, *J* = 4.8 Hz, 2H), 7.49 (t, *J* = 7.2 Hz, 2H), 7.34 (t, *J* = 7.2 Hz, 2H), 5.37 (s, 4H), 4.97 (d, *J* = 57 Hz, 6H), 3.42 (d, *J* = 110 Hz, 4H), 1.54 (s, 18H), 1.12 (bs, 6H) ppm; ^{13}C NMR (pyridine-d_5, 150 MHz): δ = 156.5, 155.9, 145.9, 140.6, 140.2, 132.9, 131.9, 131.4, 130.0, 127.6, 123.4, 123.0, 121.1, 94.8, 92.7, 80.0, 63.2, 49.1, 48.6, 42.9, 28.8, 14.3, 14.0 ppm. NMR spectra were complicated owing to tautomer mixture and correlation with solvent's signals; IR: $\tilde{\nu}$ = 3480, 2979, 2932, 2859, 1673, 1469, 1421, 1366, 1314, 1271, 1253, 1230, 1148, 1055, 983, 956, 881, 793, 763, 715, 681, 502, 443, 409 cm^{-1}; HRMS (DART+) m/z: $[M+NH_4]^+$: calcd. for $C_{40}H_{52}N_3O_6$: 670.3851; found 670.3851, correct isotope distribution; elemental analysis calcd. for $C_{40}H_{48}N_2O_6$: C: 73.59%, H: 7.41%, N: 4.29%, found: C: 73.06%, H: 7.36%, N: 4.11%.

3. NMR Spectra

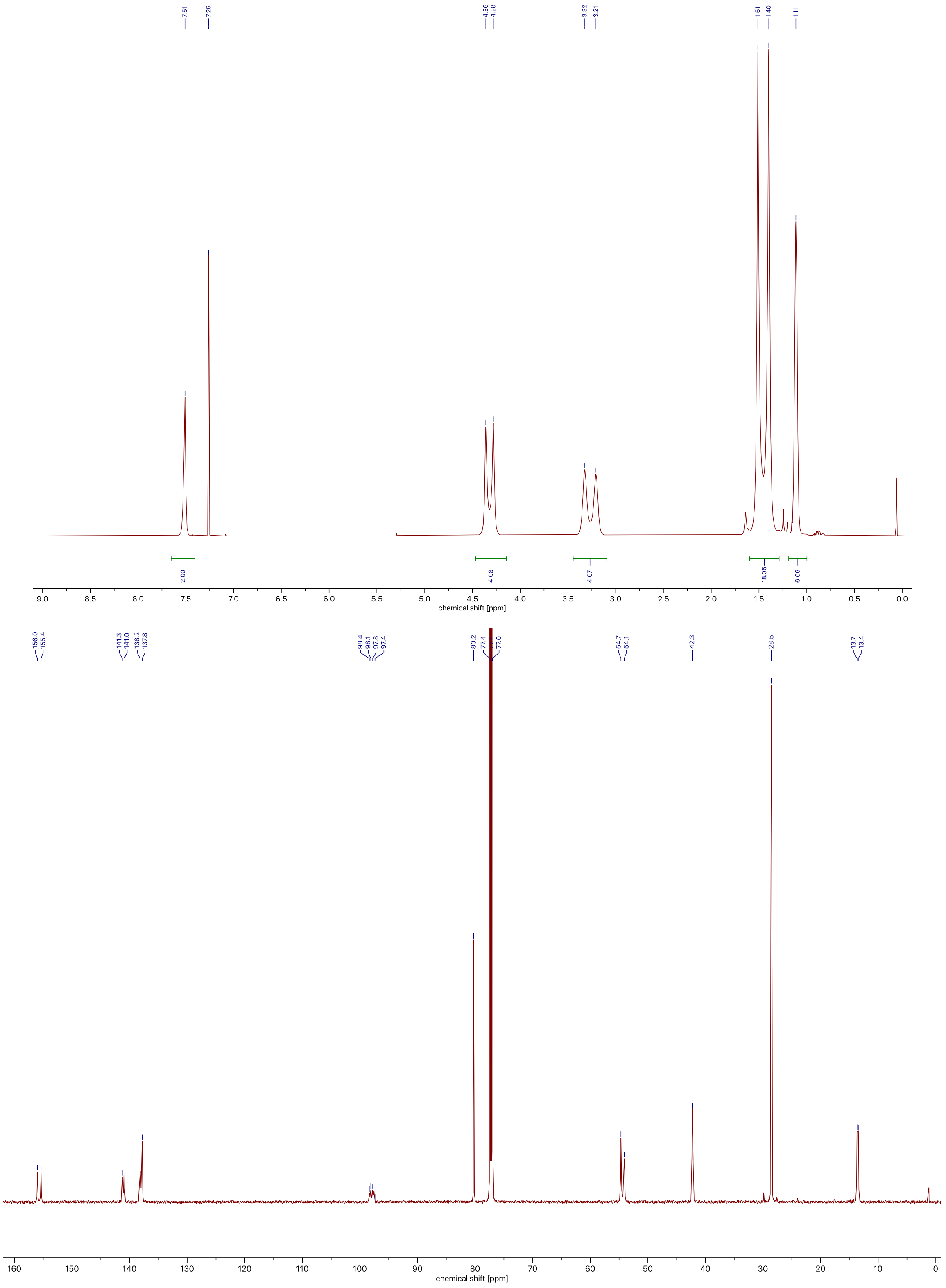

Figure S1. NMR spectra of **3**. Proton NMR spectrum (top, 600 MHz), ^{13}C NMR spectrum (bottom, 151 MHz) in $CDCl_3$.

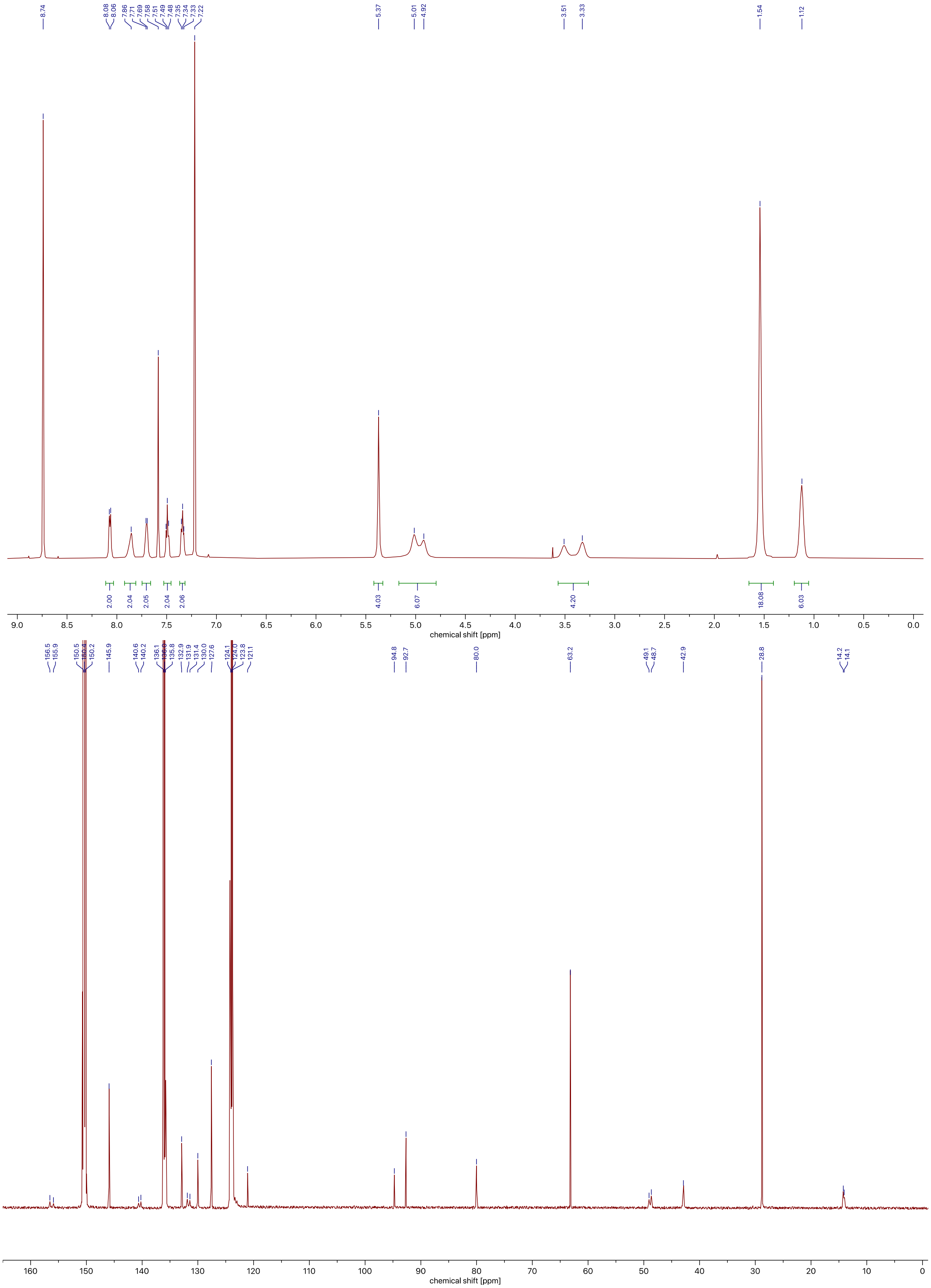

Figure S2. NMR spectra of **5**. Proton NMR spectrum (top, 600 MHz), ^{13}C NMR spectrum (bottom, 151 MHz) in pyridine-d_5.

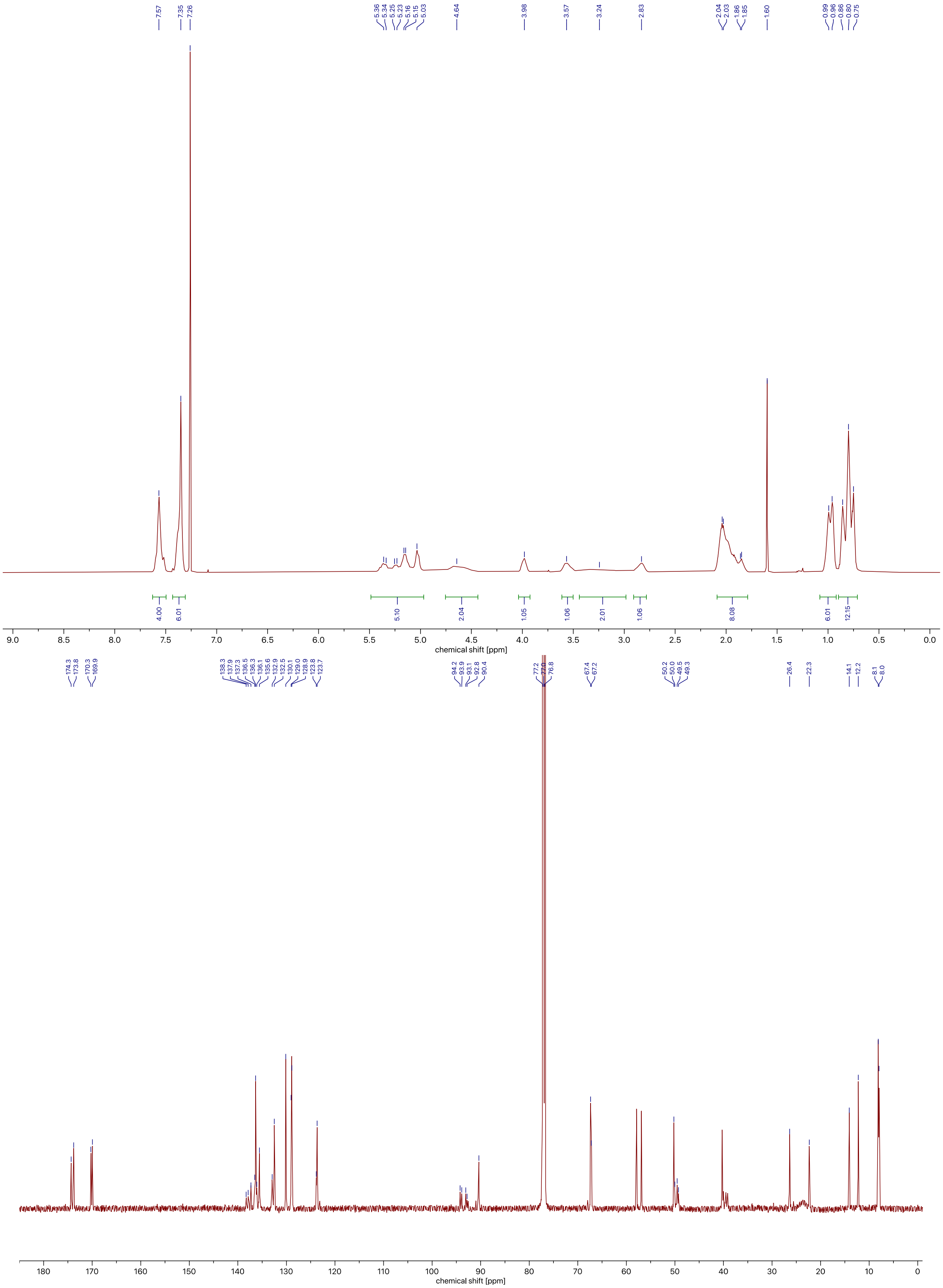

Figure S3. NMR spectra of **1**. Proton NMR spectrum (top, 600 MHz), ^{13}C NMR spectrum (bottom, 151 MHz) in $CDCl_3$.

4. Single Crystal Structure of Compound 1

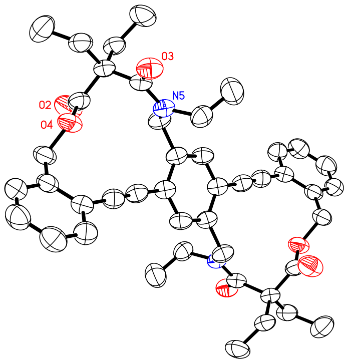

Table 1. Crystal data and structure refinement for compound **1**.

CCSD	2003158	
Empirical formula	$C_{46}H_{50}Cl_6N_2O_6$	
Formula weight	939.58	
Temperature	100(2) K	
Wavelength	1.54178 Å	
Crystal system	triclinic	
Space group	$P\bar{1}$	
Z	2	
Unit cell dimensions	a =9.6500(4) Å	α =70.925(3) deg.
	b = 15.8327(6) Å	β =85.317(3) deg.
	c = 16.2694(6) Å	γ =88.315(3) deg.
Volume	2341.39(16) $Å^3$	
Density (calculated)	1.33 g/cm^3	
Absorption coefficient	3.74 mm^{-1}	
Crystal shape	little brick	
Crystal size	0.113 x 0.057 x 0.048 mm^3	
Crystal colour	colourless	
Theta range for data collection	2.9 to 55.1 deg.	
Index ranges	-10≤h≤10, -16≤k≤16, -17≤l≤13	
Reflections collected	22606	
Independent reflections	5840 (R(int) = 0.0712)	
Observed reflections	3959 (I > 2□(I))	
Absorption correction	Semi-empirical from equivalents	
Max. and min. transmission	2.37 and 0.28	
Refinement method	Full-matrix least-squares on F^2	
Data/restraints/parameters	5840 / 677 / 640	
Goodness-of-fit on F^2	1.04	
Final R indices (I>2sigma(I))	R1 = 0.061, wR2 = 0.150	
Largest diff. peak and hole	0.28 and -0.28 $eÅ^{-3}$	

5. Computational Studies

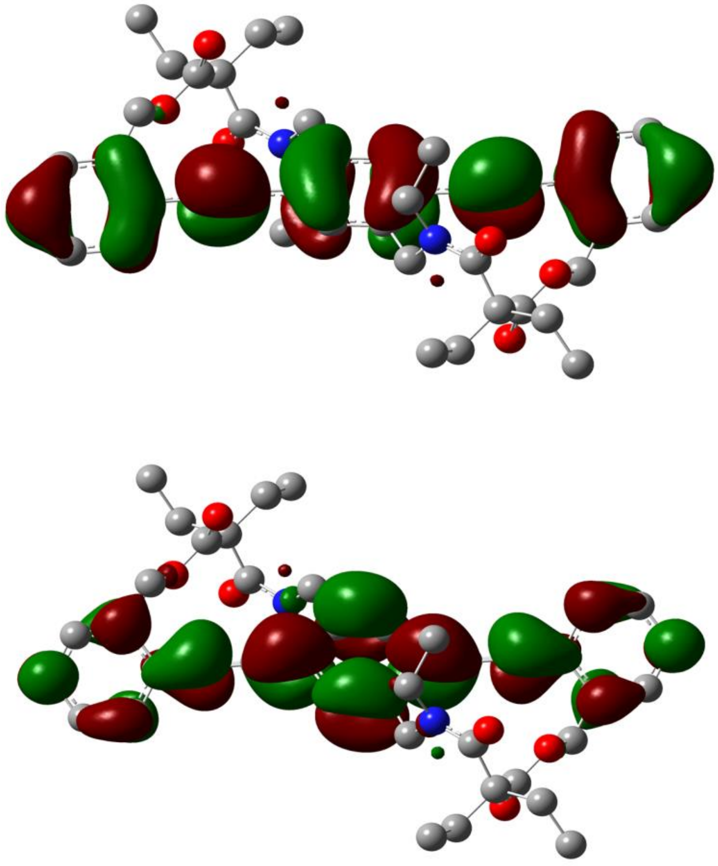

Figure S4. HOMO (top) and LUMO (bottom) of **1** calculated at the DFT/CAM-B3LYP/def2-TZVP level of theory**.**

Table 2. Vertical excitation energies *ω* and corresponding wavelengths *λ* as well as oscillator strengths *f* of the five energetically lowest singlet and triplet states of **1** calculated at the TDDFT/CAM-B3LYP/def2-TZVP level of theory at the optimized ground-state geometry.

State	*ω* [eV]	*λ* [nm]	*f*
S_1	3.83	324	1.410
S_2	4.52	274	0.000
S_3	4.62	269	0.003
S_4	4.72	263	0.002
S_5	4.92	252	0.000
T_1	2.30	538	0.000
T_2	2.94	422	0.000
T_3	3.25	382	0.000
T_4	3.83	324	0.000
T_5	4.10	303	0.000

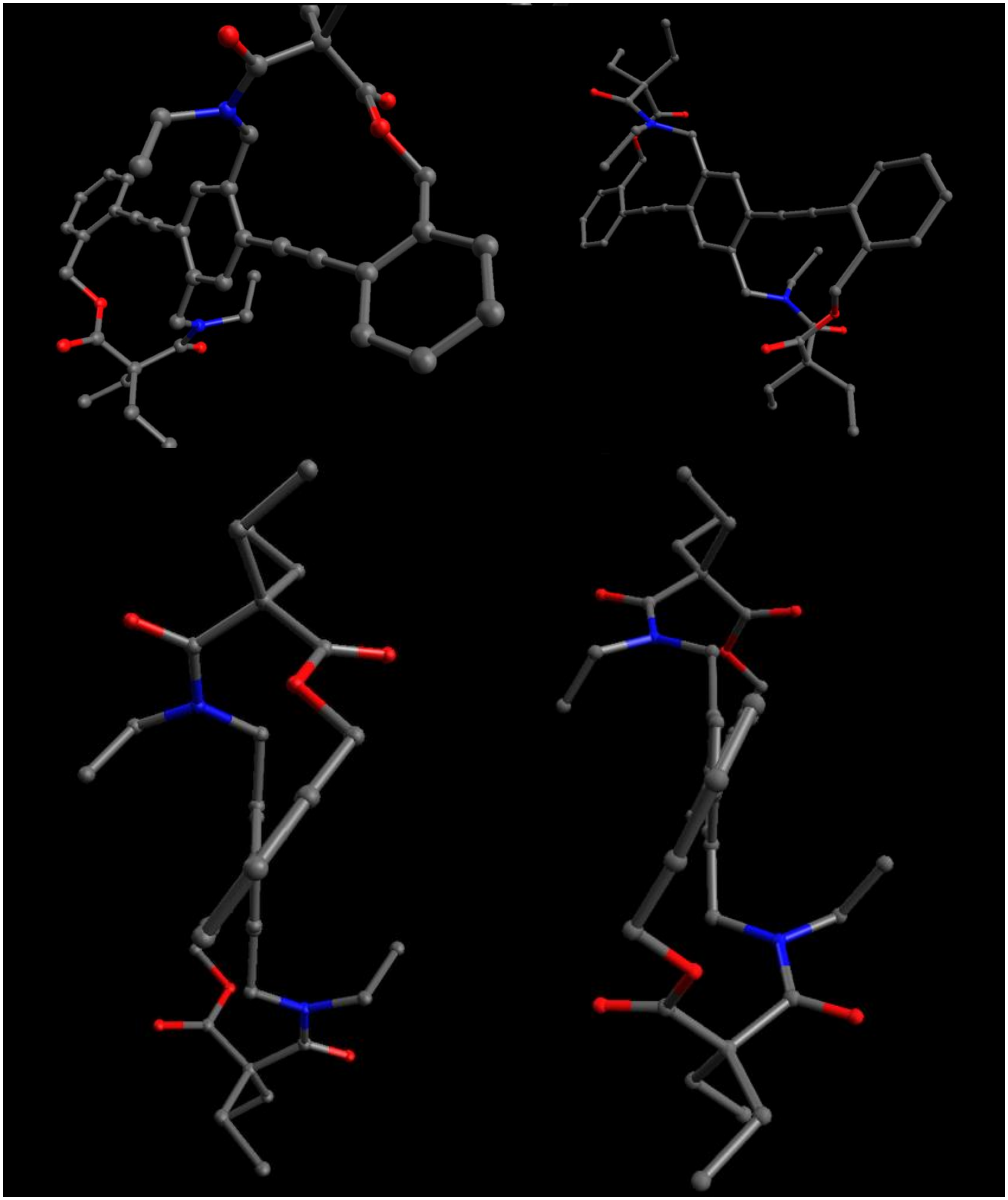

Figure S5. Comparison of different equilibrium geometries of molecule **1** in the S_0 state (left) and S_1 state (right). The bottom panel shows the view along the acetylene axis. Hydrogen atoms are omitted for clarity.

In the following, the Cartesian coordinates of molecule **1** and its corresponding dimer in equilibrium geometries of different electronic states are given.

Ground state S_0 optimized with DFT/B3LYP/def2-TZVP:
Energy: -2267.23325777 a.u.

C	-5.49190600	2.09202900	0.16196500
C	-5.40158900	0.93213000	1.15623800
O	-5.11502800	1.03286700	2.32425200
O	-5.73924300	-0.23454200	0.57909700
C	-4.64369600	1.85484000	-1.13538500
O	-5.19135200	1.96579600	-2.22027800
N	-3.28840200	1.62891300	-1.08280100
C	-6.98312000	2.17507800	-0.28307700
H	-7.07092900	2.99403600	-0.99386700
H	-7.21388000	1.27256000	-0.84509600
C	-7.99638200	2.35280500	0.84767000
H	-9.00552800	2.35670800	0.43173900
H	-7.86542500	3.29268000	1.38604800
H	-7.95145300	1.54525400	1.58207400
C	-5.04503700	3.40017800	0.86297900
H	-5.70821100	3.56409200	1.71248000
H	-4.06026800	3.25987100	1.30454100
C	-5.03083100	4.64259600	-0.02739600
H	-4.69125100	5.50490700	0.54934000
H	-6.01957300	4.88132400	-0.42046000
H	-4.35944300	4.53087900	-0.88095300
C	-2.57681000	1.77241400	-2.36816800
H	-3.02780600	2.60751200	-2.90420700
H	-1.54546400	2.03929400	-2.13986700
C	-2.61281400	0.53102000	-3.25385600
H	-2.08225100	0.73439600	-4.18707200
H	-3.64086100	0.26708100	-3.49513200
H	-2.13611700	-0.32185100	-2.77144900
C	-3.40995000	-2.14948500	-0.10830500
C	-4.56924300	-2.97059000	-0.15817800
C	-5.72727300	-2.62719700	0.57093500
C	-6.84228100	-3.45318600	0.49646600
H	-7.73182600	-3.18807200	1.05516100
C	-6.83271500	-4.60812300	-0.27802600
H	-7.71239800	-5.23708900	-0.32202600
C	-5.69168300	-4.94786000	-0.99466800
H	-5.67545400	-5.84432000	-1.60109600
C	-4.56860700	-4.13680400	-0.93575200
H	-3.67783300	-4.39457600	-1.49283000
C	-5.74170000	-1.40333200	1.43470100
H	-6.63535900	-1.37295500	2.05894000
H	-4.86941000	-1.35743000	2.08616300
C	-2.43170800	-1.44049500	-0.06996300
C	-1.23399500	-0.67956400	-0.03915800
C	-1.22097200	0.72834800	0.04484500
C	0.00993500	1.36517300	0.08768500
H	0.03972900	2.44543200	0.15226600
C	-2.47673700	1.57369000	0.13979900
H	-3.08427200	1.19625900	0.95726300
H	-2.15779700	2.58304800	0.42005100

C	5.49190500	-2.09203400	-0.16192000
C	5.40154100	-0.93215800	-1.15621600
O	5.11498900	-1.03293100	-2.32423000
O	5.73920900	0.23452800	-0.57911300
C	4.64368700	-1.85485000	1.13542500
O	5.19134000	-1.96579900	2.22032100
N	3.28839500	-1.62891800	1.08283600
C	6.98312400	-2.17501300	0.28312200
H	7.07096600	-2.99395100	0.99393100
H	7.21384800	-1.27247300	0.84512000
C	7.99639100	-2.35272600	-0.84762200
H	9.00553700	-2.35657900	-0.43169200
H	7.86547100	-3.29261900	-1.38597800
H	7.95142800	-1.54519400	-1.58204400
C	5.04509100	-3.40021700	-0.86290700
H	5.70827600	-3.56412300	-1.71240100
H	4.06031900	-3.25995800	-1.30447800
C	5.03092800	-4.64261500	0.02749500
H	4.69138600	-5.50495100	-0.54922300
H	6.01967800	-4.88129500	0.42057100
H	4.35953100	-4.53090500	0.88104600
C	2.57679600	-1.77242100	2.36819900
H	3.02778400	-2.60752600	2.90423400
H	1.54544900	-2.03929200	2.13989200
C	2.61280600	-0.53103300	3.25389500
H	2.08223700	-0.73441200	4.18710800
H	3.64085300	-0.26710300	3.49517600
H	2.13611600	0.32184400	2.77149200
C	3.40994900	2.14949500	0.10829300
C	4.56924400	2.97060000	0.15811400
C	5.72725500	2.62718400	-0.57101800
C	6.84226800	3.45317100	-0.49659800
H	7.73179900	3.18803900	-1.05530700
C	6.83272300	4.60812900	0.27786200
H	7.71241000	5.23709400	0.32182300
C	5.69171000	4.94789000	0.99452200
H	5.67549800	5.84436700	1.60092500
C	4.56863000	4.13683500	0.93565600
H	3.67787000	4.39462500	1.49274800
C	5.74165700	1.40329400	-1.43475000
H	6.63530300	1.37289400	-2.05900600
H	4.86935400	1.35737900	-2.08619300
C	2.43170700	1.44050300	0.06999000
C	1.23399400	0.67957200	0.03918900
C	1.22097100	-0.72834000	-0.04481300
C	-0.00993600	-1.36516500	-0.08765400
H	-0.03973100	-2.44542400	-0.15223600
C	2.47673500	-1.57368300	-0.13976800
H	3.08427500	-1.19624700	-0.95722600
H	2.15779400	-2.58303900	-0.42002800

First excited singlet state S_1 optimized with TDDFT/CAM-B3LYP/def2-SVP:

C	-5.46476600	-2.10121500	-0.14952100
C	-5.41954200	-0.91230800	-1.10134200
O	-5.16219300	-0.97223800	-2.27649600
O	-5.76756300	0.22562100	-0.49082900
C	-4.56424700	-1.90725600	1.10520000
O	-5.06475900	-2.02846500	2.20765500
N	-3.20805100	-1.72259200	0.99362300
C	-6.92699000	-2.19478000	0.36006700
H	-6.98180100	-3.02853000	1.07054100
H	-7.12708700	-1.29554100	0.95498700
C	-7.98566100	-2.34924700	-0.72556800
H	-8.98724700	-2.35324500	-0.27125900
H	-7.88149000	-3.28864200	-1.28808100
H	-7.96450300	-1.52521000	-1.45640600
C	-5.04948000	-3.37657100	-0.91379600
H	-5.76199900	-3.51931900	-1.73800000
H	-4.08802100	-3.20926100	-1.41408100
C	-4.97534000	-4.63909200	-0.06317400
H	-4.68138800	-5.49808900	-0.68406000
H	-5.94084700	-4.88453400	0.40192100
H	-4.23557200	-4.54844500	0.74686600
C	-2.45597900	-1.92556400	2.23855500
H	-2.91239300	-2.77455500	2.76684900
H	-1.43263000	-2.21371000	1.96411200
C	-2.43460900	-0.72047100	3.16800600
H	-1.87697900	-0.96596000	4.08484500
H	-3.45966400	-0.44847700	3.45109100
H	-1.95366400	0.14796500	2.69759400
C	-3.43805100	2.11268100	0.09506700
C	-4.55090500	2.96455000	0.15374200
C	-5.78146900	2.62409200	-0.47933000
C	-6.85863000	3.49299500	-0.37119000
H	-7.80178800	3.22986400	-0.85625900
C	-6.75567500	4.69586700	0.32899900
H	-7.61832800	5.36126200	0.39927100
C	-5.54528600	5.04621900	0.93185200
H	-5.45690300	5.98856000	1.47636600
C	-4.45637800	4.19764100	0.84596400
H	-3.51043800	4.45905900	1.32272200
C	-5.87591900	1.38858000	-1.31781400
H	-6.83777100	1.35155600	-1.85075700
H	-5.07535300	1.35666600	-2.07070100
C	-2.44258600	1.38210300	0.07893700
C	-1.26081800	0.65637700	0.06612100
C	-1.20427900	-0.77091300	-0.14947000
C	0.02513400	-1.36732700	-0.22812700
H	0.08587100	-2.44719100	-0.37712700
C	-2.45186500	-1.61611100	-0.25525400
H	-3.09693000	-1.20045900	-1.03731200
H	-2.13815900	-2.61656000	-0.59942400
C	5.46477000	2.10119900	0.14966900
C	5.41970200	0.91221900	1.10140800
O	5.16232500	0.97202800	2.27656200

O	5.76766700	-0.22566000	0.49076900
C	4.56427500	1.90722000	-1.10506600
O	5.06479800	2.02845700	-2.20751300
N	3.20807500	1.72257600	-0.99350800
C	6.92698100	2.19499400	-0.35991200
H	6.98168200	3.02881000	-1.07031900
H	7.12719600	1.29583000	-0.95490500
C	7.98563400	2.34951200	0.72573400
H	8.98721900	2.35367700	0.27142400
H	7.88134200	3.28884800	1.28832200
H	7.96458500	1.52541300	1.45650500
C	5.04931000	3.37644300	0.91403500
H	5.76179300	3.51921500	1.73826600
H	4.08786000	3.20897200	1.41428500
C	4.97502900	4.63902200	0.06351000
H	4.68095600	5.49793300	0.68445800
H	5.94051500	4.88462000	-0.40154500
H	4.23529000	4.54834500	-0.74655200
C	2.45602500	1.92553900	-2.23845500
H	2.91246400	2.77451000	-2.76676000
H	1.43267900	2.21371300	-1.96403200
C	2.43464100	0.72042800	-3.16788100
H	1.87702700	0.96591000	-4.08473100
H	3.45969300	0.44840500	-3.45094800
H	1.95367000	-0.14798800	-2.69745600
C	3.43805900	-2.11264900	-0.09510800
C	4.55090200	-2.96452000	-0.15395200
C	5.78152000	-2.62413100	0.47905400
C	6.85866200	-3.49303700	0.37075200
H	7.80186200	-3.22995800	0.85577200
C	6.75564100	-4.69584300	-0.32953800
H	7.61828200	-5.36124100	-0.39993700
C	5.54520100	-5.04612900	-0.93232800
H	5.45676600	-5.98842000	-1.47692000
C	4.45630900	-4.19754700	-0.84627700
H	3.51032800	-4.45891400	-1.32298400
C	5.87604900	-1.38869600	1.31764200
H	6.83793700	-1.35174000	1.85052600
H	5.07553400	-1.35683300	2.07058500
C	2.44259200	-1.38207600	-0.07884500
C	1.26082200	-0.65635300	-0.06601600
C	1.20428200	0.77093600	0.14957700
C	-0.02513100	1.36735100	0.22823000
H	-0.08586800	2.44721500	0.37722800
C	2.45187000	1.61613200	0.25536100
H	3.09692000	1.20049300	1.03743700
H	2.13816600	2.61659000	0.59950400

First excited triplet state T_1 optimized with TDDFT/TDA/CAM-B3LYP/def2-SVP:

C	5.46943300	2.09494900	-0.15317900
C	5.40908000	0.91302700	-1.11301800
O	5.12944800	0.97992200	-2.28271700
O	5.76793300	-0.22849200	-0.51647300
C	4.57612100	1.89690300	1.10685000
O	5.08581000	2.00299900	2.20649200
N	3.21710400	1.72723600	1.00374100
C	6.93536200	2.17708300	0.34714200
H	7.00018600	3.00697200	1.06129300
H	7.13422200	1.27376800	0.93611900
C	7.98746500	2.33063800	-0.74497600
H	8.99213100	2.32732000	-0.29752000
H	7.88463000	3.27287500	-1.30296200
H	7.95703300	1.50950800	-1.47876200
C	5.05593900	3.37829600	-0.90498700
H	5.76458500	3.52425000	-1.73203500
H	4.09093900	3.21975500	-1.40115600
C	4.99282700	4.63450100	-0.04416600
H	4.70052200	5.49989800	-0.65686000
H	5.96181800	4.87114700	0.41822500
H	4.25632800	4.54147400	0.76857900
C	2.47477800	1.93431800	2.25359400
H	2.94896900	2.77112800	2.78550800
H	1.45550700	2.24278500	1.98582200
C	2.43608000	0.72365000	3.17511100
H	1.89001100	0.97385700	4.09758300
H	3.45759900	0.42962500	3.44894600
H	1.93508700	-0.13254200	2.70321300
C	3.43984100	-2.10503300	0.10581800
C	4.56557200	-2.95593000	0.16483400
C	5.77391900	-2.62247100	-0.49972700
C	6.85879900	-3.48479400	-0.40253500
H	7.78889600	-3.22627300	-0.91425600
C	6.77760200	-4.67273600	0.32421000
H	7.64395300	-5.33390300	0.38643600
C	5.58680500	-5.01175300	0.96494300
H	5.51423000	-5.94158700	1.53257900
C	4.49123600	-4.16621400	0.88587100
H	3.55775300	-4.42151000	1.38944800
C	5.84914200	-1.38951900	-1.34726500
H	6.79615800	-1.35701600	-1.90638900
H	5.02925600	-1.35577700	-2.07831200
C	2.44472900	-1.37632100	0.08404300
C	1.26755800	-0.65540300	0.07013000
C	1.20410400	0.78858800	-0.13529400
C	-0.01823500	1.37624800	-0.21800700
H	-0.08491300	2.45654000	-0.36127200
C	2.45121600	1.63496700	-0.23916300
H	3.08909000	1.22928700	-1.03185100
H	2.13391000	2.63927000	-0.56867100
C	-5.46942200	-2.09496500	0.15321800
C	-5.40915900	-0.91303300	1.11305100
O	-5.12940100	-0.97988200	2.28272300

O	-5.76800100	0.22847600	0.51647900
C	-4.57613600	-1.89685100	-1.10682000
O	-5.08583500	-2.00294800	-2.20645800
N	-3.21712000	-1.72717700	-1.00372200
C	-6.93534800	-2.17722700	-0.34708800
H	-7.00010700	-3.00712600	-1.06123400
H	-7.13429200	-1.27393200	-0.93606800
C	-7.98742700	-2.33086800	0.74504000
H	-8.99209800	-2.32764000	0.29759400
H	-7.88450600	-3.27309300	1.30303000
H	-7.95706000	-1.50973200	1.47882300
C	-5.05581100	-3.37827200	0.90502800
H	-5.76443800	-3.52428300	1.73208300
H	-4.09082000	-3.21964500	1.40118900
C	-4.99259700	-4.63447600	0.04421300
H	-4.70021200	-5.49984400	0.65690800
H	-5.96157100	-4.87120800	-0.41816900
H	-4.25611300	-4.54138800	-0.76853800
C	-2.47480500	-1.93421000	-2.25359000
H	-2.94899800	-2.77099900	-2.78553400
H	-1.45553200	-2.24268400	-1.98583800
C	-2.43611500	-0.72350300	-3.17505600
H	-1.89005100	-0.97367100	-4.09754300
H	-3.45763500	-0.42946700	-3.44887300
H	-1.93511900	0.13267000	-2.70312500
C	-3.43986400	2.10502700	-0.10584500
C	-4.56560600	2.95590900	-0.16489500
C	-5.77395300	2.62245800	0.49966900
C	-6.85883800	3.48477200	0.40245300
H	-7.78893500	3.22625700	0.91417600
C	-6.77764700	4.67269400	-0.32432500
H	-7.64400300	5.33385300	-0.38657300
C	-5.58685100	5.01170200	-0.96506300
H	-5.51428000	5.94152200	-1.53272300
C	-4.49127500	4.16617400	-0.88596200
H	-3.55779100	4.42146500	-1.38954000
C	-5.84917400	1.38952800	1.34724100
H	-6.79618100	1.35705300	1.90638200
H	-5.02927700	1.35579400	2.07827500
C	-2.44473600	1.37633800	-0.08405700
C	-1.26756100	0.65542500	-0.07010100
C	-1.20410800	-0.78856600	0.13531900
C	0.01823100	-1.37622600	0.21803600
H	0.08490800	-2.45652000	0.36128800
C	-2.45122000	-1.63494400	0.23917700
H	-3.08908600	-1.22928000	1.03188100
H	-2.13391600	-2.63925600	0.56865800

Ground state S_0 of the corresponding dimer optimized with DFT/B3LYP/def2-TZVP:

C	-0.00719400	2.53621100	-0.09161600
C	0.72820500	1.65746100	-1.10580800
O	0.56977700	1.68768900	-2.30189500
O	1.63505400	0.86875700	-0.50309900
C	-0.66074500	1.71669300	1.07461800
O	-0.41129300	2.03882900	2.22555700
N	-1.57813000	0.72234400	0.83437400
C	1.07216500	3.45697300	0.55371400
H	0.57242700	4.09208100	1.28207700
H	1.74722200	2.82618700	1.12842200
C	1.87435600	4.31434900	-0.42534200
H	2.62474000	4.88526200	0.12463900
H	1.25257100	5.03184000	-0.96289800
H	2.40356500	3.71612900	-1.17065500
C	-1.08511900	3.37082200	-0.82897400
H	-0.57846000	3.97859000	-1.57874700
H	-1.73085100	2.70733100	-1.40114300
C	-1.94103600	4.26524900	0.06761500
H	-2.67756000	4.79670800	-0.53796300
H	-1.34730400	5.01466600	0.59207900
H	-2.48407600	3.69353400	0.82255900
C	-2.35211400	0.28354100	2.01179700
H	-2.56718100	1.16640800	2.61398400
H	-3.29898000	-0.11320100	1.64767400
C	-1.64924700	-0.75561800	2.87980900
H	-2.28433300	-1.00657300	3.73289900
H	-0.70850000	-0.36006300	3.25839600
H	-1.44692100	-1.67165500	2.32558700
C	0.89869400	-2.11685200	-0.16700700
C	2.30645700	-2.07436700	0.02895400
C	3.08134500	-1.03718600	-0.53134800
C	4.45455900	-1.02742300	-0.31756200
H	5.04818500	-0.22917200	-0.74677900
C	5.07397600	-2.02096700	0.43257500
H	6.14492200	-1.99417600	0.58681700
C	4.31154800	-3.04387100	0.98334600
H	4.78442300	-3.82112300	1.56991600
C	2.93960200	-3.07124900	0.78383700
H	2.34036400	-3.86394100	1.21144100
C	2.43308600	0.02329400	-1.36745800
H	3.17949900	0.64356400	-1.86484000
H	1.78242800	-0.40429100	-2.12990100
C	-0.29866800	-2.14083600	-0.32746400
C	-1.70471200	-2.25520900	-0.50762000
C	-2.55578100	-1.13089900	-0.57397700
C	-3.91751300	-1.34473400	-0.77861500
H	-4.57534100	-0.48597900	-0.84198800
C	-2.05271000	0.29750600	-0.49053700
H	-1.25270800	0.42485700	-1.21412700
H	-2.87804400	0.94138100	-0.81167200
C	-4.45186300	-2.62090000	-0.90262500
C	-3.61381200	-3.72594200	-0.82162600
C	-2.25521500	-3.54211100	-0.62731700

H	-1.59126000	-4.39451400	-0.57346200
H	-5.51492700	-2.74910700	-1.06079400
H	-4.01487600	-4.72697400	-0.91485200

First excited singlet state S_1 of the corresponding dimer optimized with TDDFT/CAM-B3LYP/def2-SVP:

C	-0.40893100	2.50740400	-0.07620900
C	0.51441100	1.76247900	-1.03291000
O	0.41458000	1.76248700	-2.23338300
O	1.51260100	1.15213700	-0.38513300
C	-0.98767800	1.59466500	1.04278900
O	-0.83844800	1.92793700	2.20411900
N	-1.75092200	0.49272900	0.74702200
C	0.47043200	3.57424700	0.62811500
H	-0.16299600	4.10926800	1.34609000
H	1.21016200	3.04125700	1.23762900
C	1.17109600	4.55839400	-0.30130800
H	1.80760700	5.23639500	0.28589500
H	0.46433400	5.18479300	-0.86511300
H	1.82168700	4.05671100	-1.03516100
C	-1.55229500	3.16037400	-0.88204200
H	-1.09952000	3.86226300	-1.59588300
H	-2.03426400	2.40531900	-1.51468300
C	-2.60310600	3.87572700	-0.04120700
H	-3.37188400	4.31881100	-0.69119400
H	-2.17174500	4.68909800	0.55975400
H	-3.11267000	3.19065600	0.65357900
C	-2.50394500	-0.05459700	1.88303300
H	-2.88415700	0.79349600	2.47045000
H	-3.36817600	-0.59595800	1.47677900
C	-1.69458300	-0.96386300	2.79714100
H	-2.32311400	-1.30750700	3.63309100
H	-0.84119400	-0.41287300	3.21288800
H	-1.32199800	-1.84865700	2.26342700
C	1.25752700	-1.89062800	-0.20273000
C	2.60870900	-1.70390900	-0.01541500
C	3.27442300	-0.48481100	-0.42029200
C	4.62520200	-0.34006700	-0.15781100
H	5.12538400	0.58382700	-0.45918400
C	5.36741900	-1.35252600	0.45951500
H	6.43239700	-1.20784800	0.65071200
C	4.74235800	-2.55841500	0.82065900
H	5.32410400	-3.35278400	1.29293100
C	3.39807400	-2.73922400	0.59275100
H	2.90018300	-3.66512800	0.88520300
C	2.52612200	0.54278100	-1.19904500
H	3.20446200	1.33155500	-1.55684900
H	2.03626900	0.09586600	-2.07724300
C	0.02283000	-2.10046900	-0.32337700
C	-1.30287000	-2.44059200	-0.46175000
C	-2.35418700	-1.46785700	-0.67556100
C	-3.63684300	-1.92908000	-0.92264500
H	-4.43087100	-1.19680300	-1.09207600
C	-2.08848700	0.01337200	-0.59828400

H	-1.27222400	0.26891000	-1.28512300
H	-2.99194000	0.52639300	-0.97005800
C	-3.95305800	-3.29160800	-0.94087000
C	-2.95294600	-4.24028400	-0.67007800
C	-1.66334800	-3.83428700	-0.42153800
H	-0.87626000	-4.56395200	-0.22477800
H	-4.97703700	-3.61156700	-1.14194500
H	-3.19790800	-5.30450400	-0.65870900

Energetically higher conformer of the bridged trimer **1** optimized at the DFT/B3LYP/def2-TZVP level of theory: (Energy: -2267.22536013 a.u., thus 0.00789757 a.u. (0.2149033 eV, 20.73507 kJ/mol) higher than the first geometry. Hence, the conformer is not expected to be populated at ambient temperature.)

C	5.279894	-2.169484	0.699870
C	5.299609	-0.834063	1.449640
O	5.217067	-0.687751	2.644518
O	5.454833	0.186666	0.589415
C	4.217698	-2.122707	-0.451098
O	4.549763	-2.463983	-1.575595
N	2.940410	-1.675443	-0.212630
C	6.673263	-2.327368	0.020676
H	6.656957	-3.247292	-0.559882
H	6.784208	-1.524239	-0.704975
C	7.866891	-2.327103	0.975550
H	8.792287	-2.384450	0.399488
H	7.858543	-3.177408	1.659210
H	7.916667	-1.419121	1.581095
C	5.012807	-3.329163	1.690178
H	5.793901	-3.303646	2.450399
H	4.088839	-3.143670	2.233921
C	4.954081	-4.718436	1.054968
H	4.712776	-5.462677	1.816226
H	5.904913	-5.007321	0.605991
H	4.193753	-4.782007	0.274209
C	2.086655	-1.579586	-1.411146
C	3.525611	1.972824	-1.068122
C	4.758881	2.684672	-1.018957
C	5.686913	2.491936	0.026329
C	6.868769	3.225107	0.023979
H	7.580728	3.074525	0.826605
C	7.150022	4.141616	-0.982461
H	8.076619	4.700635	-0.962225
C	6.237150	4.332029	-2.012186
H	6.445672	5.041615	-2.802684
C	5.053154	3.610855	-2.029907
H	4.338327	3.753975	-2.829140
C	5.412291	1.523826	1.138201
H	6.155459	1.610733	1.929758
H	4.425386	1.680398	1.577662
C	2.465820	1.395452	-1.138561
C	1.170036	0.812513	-1.223414
C	0.958186	-0.580029	-1.322146
C	-0.348374	-1.029812	-1.414551
H	-0.530631	-2.093262	-1.501560

C	-4.365063	1.536688	1.849129
C	-5.633563	0.909591	1.275935
O	-6.758874	1.303496	1.465845
O	-5.358760	-0.191926	0.547840
C	-3.276690	1.959770	0.805590
O	-2.149522	2.161199	1.231714
N	-3.520362	2.062895	-0.538979
C	-3.760445	-1.444687	-1.459330
C	-4.850352	-2.360518	-1.413789

6. Comparison of Optical Spectra

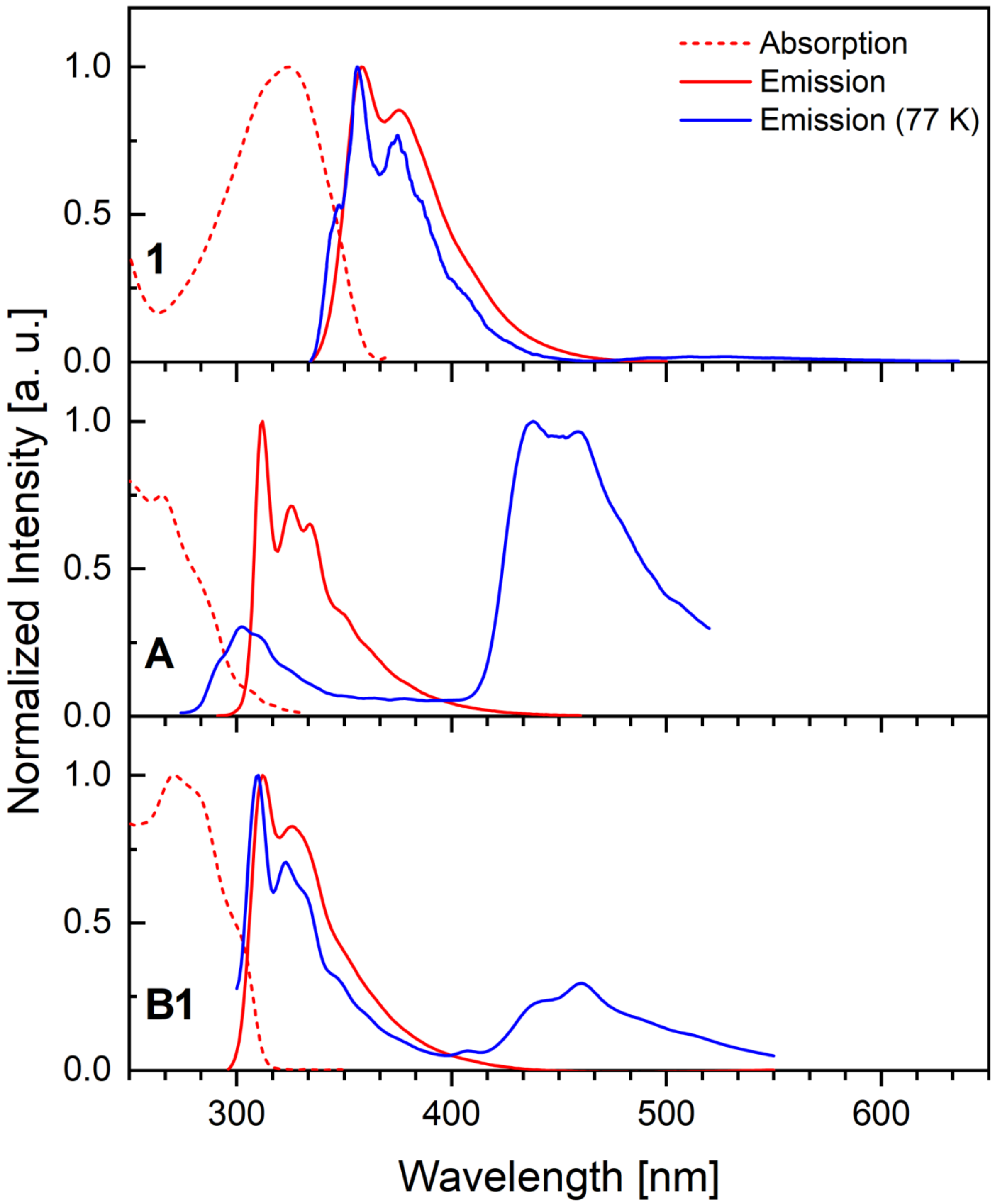

Figure S6. Comparison of absorption and emission spectra of 1, A and B1. UV-Vis absorption (dashed) and emission spectrum (solid) in *n*-hexane at room temperature (red) and emission spectrum in a frozen EPA (Et_2O/isopentane/EtOH 5:2:2 v/v/v) glass (blue) at 77 K.[6]

7. Illustration of Excited States of BPEP

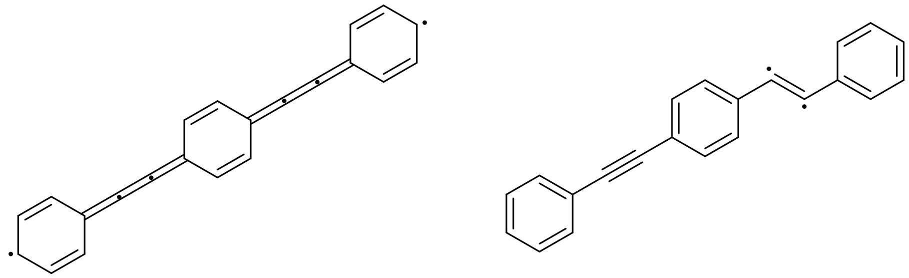

Chart C1. Schematic illustration of the first excited state with a chinoidal-cumulenic structure (left) or of its dark state ("*trans*-bent", right).

8. References

[1] G. R. Fulmer, A. J. M. Miller, N. H. Sherden, H. E. Gottlieb, A. Nudelman, B. M. Stoltz, J. E. Bercaw, K. I. Goldberg, *Organometallics* **2010**, *29*, 2176-2179.

[2] U. Resch-Genger, K. Rurack, *Pure Appl. Chem.* **2013**, *85*, 2005-2026.

[3] A. M. Brouwer, *Pure Appl. Chem.* **2011**, *83*, 2213-2228.

[4] G. Gaefke, V. Enkelmann, S. Höger, *Synthesis* **2006**, *2006*, 2971–2973.

[5] H. Wu, Y.-P. He, L.-Z. Gong, *Org. Lett.* **2013**, *15*, 460-463.

[6] a) Y. Kozhemyakin, M. Krämer, F. Rominger, A. Dreuw, U. H. F. Bunz, *Chem. Eur. J.* **2018**, *24*, 15219–15222; b) S. Menning, M. Krämer, B. A. Coombs, F. Rominger, A. Beeby, A. Dreuw, U. H. F. Bunz, *J. Am. Chem. Soc.* **2013**, *135*, 2160–2163.
